# Do clinical trials truly mirror their target population? An external validity analysis of national register versus trial data from the Swedish prospective SENOMIC trial on sentinel node micrometastases in breast cancer

**DOI:** 10.1007/s10549-019-05328-3

**Published:** 2019-06-24

**Authors:** Y. Andersson, L. Bergkvist, J. Frisell, J. de Boniface

**Affiliations:** 1 Department of Surgery, Västmanland County Hospital, SE- 72189 Västerås, Sweden; 2 Center for Clinical Research, Uppsala University, Västmanland County Hospital, Västerås, Sweden; 3 Department of Breast and Endocrine Surgery, Karolinska University Hospital, Stockholm, Sweden; 4 Department of Molecular Medicine and Surgery, Karolinska Institutet, Stockholm, Sweden; 5 Department of Surgery, Capio St Göran’s Hospital, Stockholm, Sweden

**Keywords:** Breast cancer, Sentinel node biopsy, Micrometastases, Axillary lymph node dissection, Cohort study, Register

## Abstract

**Purpose:**

Increasing evidence suggests that completion axillary lymph node dissection (ALND) may be omitted in breast cancer patients with limited axillary nodal metastases. However, the representativeness of trial participants for the original clinical practice population, and thus, the generalizability of published trials have been questioned. We propose the use of background data from national registers as a means to assess whether trial participants mirror their target population and to strengthen the generalizability and implementation of trial outcomes.

**Methods:**

The Swedish prospective SENOMIC trial, omitting a completion ALND in breast cancer patients with sentinel lymph node micrometastases, reached full target accrual in 2017. To assess the generalizability of trial results for the target population, a comparative analysis of trial participants versus cases reported to the Swedish National Breast Cancer Register (NKBC) was performed.

**Results:**

Comparing 548 trial participants and 1070 NKBC cases, there were no significant differences in age, tumor characteristics, breast surgery, or adjuvant treatment. Only the mean number of sentinel lymph nodes with micrometastasis per individual was lower in trial participants than in register cases (1.06 vs. 1.09, *p* = 0.037).

**Conclusions:**

Patients included in the SENOMIC trial are acceptably representative of the Swedish breast cancer target population. There were some minor divergences between trial participants and the NKBC population, but taking these into consideration, upcoming trial outcomes should be generalizable to breast cancer patients with micrometastases in their sentinel lymph node biopsy.

## Introduction

Sentinel lymph node biopsy (SLN biopsy) is the standard axillary staging procedure in breast cancer and in SLN-negative patients, completion axillary lymph node dissection (ALND) is omitted without compromising oncological safety [1–4]. An increasing number of publications suggest that completion ALND may be omitted also in case of limited nodal metastasis [5–9]. The randomized ACOSOG Z0011 and IBCSG 23-01 trials found no survival benefit in SLN-positive patients who had completion ALND compared with those who had SLN biopsy only [10–12]. This had a major impact on clinical practice: In many countries, completion ALND is today omitted in patients with breast-conserving surgery and micrometastases (≤ 2 mm) or fewer than two macrometastases (> 2 mm) in their SLN.

However, the generalizability of results has been questioned [13–16]. Both ACOSOG Z0011 and IBCSG 23-01 closed early due to slow accrual and low event rates, and the majority of included patients had favorable prognostic tumor characteristics [17, 18]. Furthermore, very few patients with a mastectomy were included.

The tendency to select the healthiest patients in clinical trials has been discussed in a review by Ford and Norrie in 2016 [19]. The authors propose the concept of pragmatic trials in order to increase external validity. This includes, e.g., broadening inclusion criteria, striving to acquire a study population that is as close as possible to the clinical practice population to whom the trial results will be applied to in the end.

With the purpose of confirming the safety of ALND omission in the case of SLN micrometastases also in patients with less favorable prognostic characteristics and patients who undergo mastectomy, we performed a Swedish prospective cohort study, the SENOMIC trial, the oncological outcomes of which will be reported in due course.

The Swedish National Breast Cancer Register contains information on age, gender, primary tumor and lymph node characteristics, surgical intervention, adjuvant, and neoadjuvant treatment and follow-up data. The coverage rate for primary breast cancer cases is 98–99% [20]. With the intention to explore the representativeness of SENOMIC participants, and thus assess the external validity, these were compared with eligible cases reported to the Swedish National Breast Cancer Register (NKBC). The primary aim was to evaluate how accurately the SENOMIC sample reflects the total Swedish population of breast cancer patients with SLN micrometastases, thus comparing the SENOMIC participants with all NKBC cases. The secondary aim was to explore how patients that were included in the study differed from those who were not, by comparing SENOMIC participants with NKBC cases after exclusion of cases corresponding to SENOMIC participants.

## Patients and methods

Between January 2014 and March 2017, 566 primary breast cancer patients with SLN micrometastases from 23 Swedish hospitals were included in the prospective SENOMIC trial. The participating sites comprised both university and non-university public hospitals, and one private hospital. Patients with clinically node-negative invasive breast cancer underwent SLN biopsy together with either breast-conserving surgery or mastectomy. In case of SLN micrometastasis, patients could be offered enrolment in the SENOMIC trial, omitting completion ALND. Exclusion criteria were preoperatively diagnosed axillary lymph node metastasis, history of a previous invasive breast cancer, metastasis outside the ipsilateral axilla, SLN metastasis > 2 mm, pregnancy, and medical contraindication for adjuvant systemic treatment. At trial initiation, tumor size > 5 cm and neoadjuvant treatment were further exclusion criteria. Even though a protocol amendment allowed the inclusion of T3 tumors and of patients started on neoadjuvant treatment after their SLN biopsy from January 2017, these cases were not included in the current analysis.

The SENOMIC trial protocol states that adjuvant treatment should be given according to national and regional treatment guidelines, based on tumor and lymph node characteristics. According to national guidelines, radiotherapy to regional lymph node basins should not be given in case of micrometastases only.

Patient and tumor characteristics were validated by scrutinizing each participant’s pathology reports and were recorded together with data on adjuvant and neoadjuvant treatment. Trial participants are followed by annual clinical examinations and mammography for 5 years and then again after 10 and 15 years.

The trial was approved by the respective regional Ethical Committees and the Central Ethical Committee in Stockholm (no. 2013/1258-31/4). Data management followed the respective applicable Swedish and European legislation.

### The Swedish National Breast Cancer Register

Data on breast cancer patients with SLN micrometastases who were diagnosed between January 1, 2014 and March 31, 2017 were extracted from the Swedish National Breast Cancer Register (NKBC). Importantly, registered adjuvant treatment data represent postoperative multidisciplinary conference recommendations, not completed therapy. In accordance with trial eligibility criteria as outlined above, cases of T3 tumors and patients receiving neoadjuvant treatment were excluded, as well as cases lacking relevant information. In the NKBC data, adjuvant radiotherapy data lack definition of the clinical target volume, i.e., it is not specified whether regional lymph nodes were included in the radiation field or not.

### Statistical analysis

Patient and tumor characteristics are presented as distributions with their percentages (categorical data) or median values with their range (continuous data). The distribution of categorical data was tested by the Chi-square test and of continuous data by the Student’s *t* test. In a first step, trial participants were compared with the entire NKBC population, allowing an overlap between trial and NKBC populations. In a second step, NKBC cases corresponding to SENOMIC participants were excluded and thus, the overlap was eliminated. As NKBC data were de-identified, corresponding cases were identified by agreement of the date of surgery at the same site, as well as age and tumor size.

The statistical analysis was performed using SPSS software (IBM Corp., Armonk, N.Y., USA) version 24, and statistical significance was set at *p *< 0.05 for all tests.

## Results

**Fig. 1:**
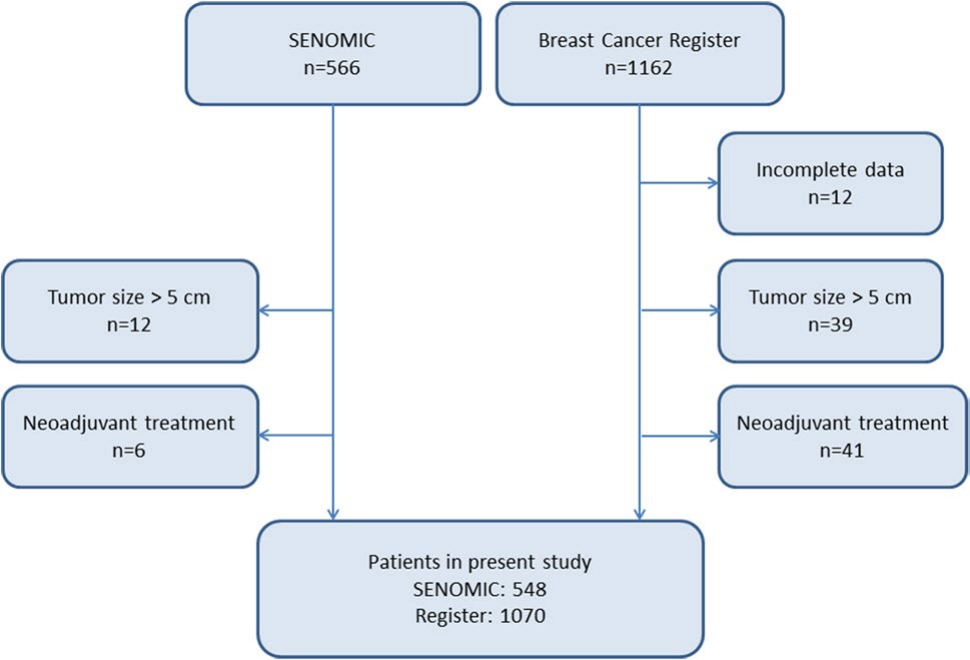
Flowchart for inclusion and exclusion of trial participants and NKBC cases

Between January 2014 and March 2017, 566 patients were included in the SENOMIC trial and 1162 new eligible breast cancer cases reported to the Swedish National Breast Cancer Register (NKBC). After the exclusion of individuals with tumor size > 5 cm, those receiving neoadjuvant treatment, and NKBC cases with incomplete data, 548 SENOMIC and 1070 NKBC patients remained for analysis (Fig. 1).

There were 23 sites participating in the SENOMIC trial and 39 sites reporting to NKBC. Based on the number of cases registered in the NKBC and fulfilling SENOMIC eligibility criteria, participating sites included between 23 and 100% (median 70%) of their patients with SLN micrometastases in the SENOMIC trial. There were no differences in inclusion rates between university and non-university hospitals.

When comparing SENOMIC participants with all NKBC cases, thus allowing an overlap between the two groups, age, gender, tumor characteristics, breast surgery, and adjuvant treatment were similar in both groups (Tables 1 and 2). The mean number of SLNs with micrometastases was lower in SENOMIC participants than in all NKBC cases (1.06 vs. 1.09, *p* = 0.037).

**Table 1:** Patient and tumor characteristics in trial participants and NKBC cases

Characteristics	Included in SENOMIC (*n *= 548)	NKBC (*n *= 1070)	*P*^e^
Age (years)^a^	62 (28–90)	62 (27–95)	0.952
Tumor size (mm)^b^	18 (8)	19 (9)	0.105
Tumor size (mm)^c^			0.335
< 20	344 (62.8)	648 (60.6)	
20–29	147 (26.8)	284 (26.5)	
30–50	57 (10.4)	138 (12.9)	
Multifocality^c^			0.548
Yes	128 (23.3)	260 (24.3)	
No	420 (76.7)	792 (74.0)	
Missing	0 (0)	18 (1.7)	
Histological tumor type^c^			0.994
Ductal	448 (81.7)	874 (81.7)	
Lobular	63 (11.5)	122 (11.4)	
Mixed	12 (2.2)	27 (2.5)	
Other	25 (4.6)	47 (4.4)	
Tumor grade (NHG)^c^			0.802
1	89 (16.2)	171 (16.0)	
2	311 (56.8)	577 (53.9)	
3	144 (26.3)	290 (27.1)	
Missing	4 (0.7)	32 (3.0)	
Estrogen receptor status^c^			0.966
Positive	499 (91.1)	970 (90.7)	
Negative	490 (8.9)	96 (9.0)	
Missing	0 (0)	4 (0.3)	
Progesterone receptor status^c^			0.695
Positive	434 (79.2)	853 (79.8)	
Negative	113 (20.6)	211 (19.7)	
Missing	1 (0.2)	6 (0.5)	
HER-2 status^c^			0.226
Amplified^d^	61 (11.1)	140 (13.1)	
Not amplified	479 (87.4)	902 (84.3)	
Missing	8 (1.5)	28 (2.6)	
Number of micrometastatic SLNs^a^	1 (1–3)	1 (1–6)	0.037
Number of SLNs^a^	2 (1–10)	2 (1–9)	0.372

*NKBC* Swedish National Breast Cancer Register, *SLNs* Sentinel lymph nodes

^a^Median (range)

^b^Mean (standard deviation)

^c^Number (%)

^d^HER-2 3 + and/or ISH positive

^e^Missing numbers excluded

**Table 2:** Given (SENOMIC) and planned (NKBC) treatment in trial participants versus register cases

Treatment	Included in SENOMIC (*n *= 548)	NKBC (*n *= 1070)	*p*
Endocrine therapy^a^	497 (90.5)	950 (88.8)	0.301
Radiotherapy^ab^	396 (72.1)	747 (69.8)	0.346
Chemotherapy^a^	290 (52.9)	544 (50.8)	0.495
Trastuzumab^a^	56 (10.2)	124 (11.6)	0.407
Breast surgery^a^			0.420
Mastectomy	204 (37.2)	420 (39.3)	
Breast conservation	344 (62.8)	649 (60.6)	
Missing	0 (0)	1 (0.1)	
Axillary surgery^a^			< 0.001
SLN biopsy only	548 (100)	853 (79.7)	
SLN biopsy and subsequent ALND	0 (0)	217 (20.3)	

*NKBC* Swedish National Breast Cancer Register, *SLN* Sentinel lymph node, *ALND* Axillary lymph node dissection

^a^Number (%)

^b^To remaining breast/chest wall and/or regional lymph nodes

In a second step, those 486 out of 1071 NKBC cases who corresponded to SENOMIC participants were excluded, leaving 585 NKBC cases for comparative analysis. For 62 SENOMIC participants, no corresponding NKBC case could be identified. Trial participants had smaller primary tumors and a lower mean number of SLNs with micrometastases (1.06 vs. 1.11, *p* = 0.003) than the 585 NKBC cases (Table 3). Furthermore, the mastectomy rate was lower in SENOMIC participants than in NKBC cases, but the difference did not reach statistical significance (Table 4).

**Table 3:** Patient and tumor characteristics in trial participants and NKBC cases, excluding cases identical to trial participants

Characteristics	Included in SENOMIC (*n *= 548)	NKBC Excluding SENOMIC participants (*n *= 585)	*P*^e^
Age (years)^a^	62 (28–90)	63 (28–95)	0.742
Tumor size (mm)^b^	18 (8)	19 (10)	0.026
Tumor size (mm)^c^			0.052
< 20	344 (62.8)	344 (58.8)	
20–29	147 (26.8)	152 (26.0)	
30–50	57 (10.4)	89 (15.2)	
Multifocality^c^			0.408
Yes	128 (23.3)	1471 (25.2)	
No	420 (76.7)	430 (73.8)	
Missing	0 (0)	6 (1.0)	
Histological tumor type^c^			0.921
Ductal	448 (81.7)	482 (82.4)	
Lobular	63 (11.5)	60 (10.3)	
Mixed	12 (2.2)	16 (2.7)	
Other	25 (4.6)	27 (4.6)	
Tumor grade (NHG)^c^			0.655
1	89 (16.2)	101 (17.4)	
2	311 (56.8)	310 (53.4)	
3	144 (26.3)	158 (27.2)	
Missing	4 (0.7)	12 (2.0)	
Estrogen receptor status^c^			0.844
Positive	499 (91.1)	528 (90.3)	
Negative	490 (8.9)	54 (9.2)	
Missing	0 (0)	3 (0.5)	
Progesterone receptor status^c^			0.828
Positive	434 (79.2)	464 (79.3)	
Negative	113 (20.6)	117 (20.0)	
Missing	1 (0.2)	4 (0.7)	
HER-2 status^c^			0.350
Amplified^d^	61 (11.1)	75 (12.8)	
Not amplified	479 (87.4)	496 (84.8)	
Missing	8 (1.5)	14 (2.4)	
Number of micrometastatic SLNs^a^	1 (1–3)	1 (1–6)	0.003
Number of SLNs^a^	2 (1–10)	2 (1–9)	0.539

*NKBC* Swedish National Breast Cancer Register, *SLNs* Sentinel lymph nodes

^a^Median (range)

^b^Mean (standard deviation)

^c^Number (%)

^d^HER-2 3 + and/or ISH positive

^e^Missing numbers excluded

**Table 4:** Given (SENOMIC) and planned (NKBC) treatment in trial participants versus register patients, excluding cases identical to trial participants

Treatment	Included in SENOMIC (*n *= 548)	NKBC Excluding SENOMIC participants (*n *= 585)	*p*
Endocrine therapy^a^	497 (90.5)	515 (88.0)	0.245
Radiotherapy^ab^	396 (72.1)	400 (68.4)	0.239
Chemotherapy^a^	290 (52.9)	291 (49.7)	0.062
Trastuzumab^a^	56 (10.2)	65 (11.1)	0.627
Breast surgery^a^			0.055
Mastectomy	204 (37.2)	255 (43.6)	
Breast conservation	344 (62.8)	329 (56.2)	
Missing	0 (0)	1 (0.2)	
Axillary surgery^a^			< 0.001
SLN biopsy only	548 (100)	365 (62.4)	
SLN biopsy and subsequent ALND	0 (0)	220 (37.6)	

*NKBC* Swedish National Breast Cancer Register, *SLN* Sentinel lymph node, *ALND* Axillary lymph node dissection

^a^Number (%)

^b^To remaining breast/chest wall and/or regional lymph nodes

Overall, completion ALND was performed in 20.3% of all patients with SLN micrometastases reported to the NKBC. When excluding NKBC cases who corresponded to SENOMIC participants, the completion ALND rate was 37.6%. Showing changes in axillary management over time, this proportion decreased from 44.6% in 2014 to 28.4% in 2016/2017 (*p* = 0.001).

## Discussion

This external validity analysis indicates good generalizability of the upcoming results of the prospective SENOMIC trial, omitting completion ALND in the case of SLN micrometastases. Apart from a small difference in the number of SLN micrometastases, SENOMIC participants reflect the NKBC population well. However, when comparing SENOMIC participants with NKBC cases without duplicates, there were also minor differences in primary tumor size and mastectomy rate. This should be considered when implementing the results, acknowledging that the NKBC population without patients corresponding to SENOMIC participants is a mixture of patients that could have been included and those that fulfilled some exclusion criterium due to factors not available in the register.

Results indicating the oncological safety of omitting ALND in SLN-positive patients from previous trials such as ACOSOG Z0011 and IBCG 23-01 [11, 22] have had major impact on clinical practice, and completion ALND has been widely abandoned. Despite that the generalizability in previous studies has been questioned, however, the indications for omitting completion ALND have subsequently been broadened to include patients not represented in any trials, those with less favorable prognostic characteristics, or those undergoing mastectomy [23]. In order not to risk undertreatment of underrepresented subgroups, it is important to ensure external validity and that trial outcomes are generalizable to patients that are treated in the general clinical setting.

Even though the SENOMIC trial had the same eligibility criteria regarding tumor size as both ACOSOG Z0011 and IBCSG 23-01 (≤ 5 cm), fewer T1 tumors were enrolled in SENOMIC (63%) than in the earlier trials (69% in ACOSOG Z0011 and IBCSG 23-01) [10, 17]. Furthermore, SENOMIC enrolled fewer grade 1 tumors (16%) than the previous two trials (24% in ACOSOG Z0011 and 22% in IBCSG 23-01), suggesting a selection bias in the latter two.

A limitation with this external validation analysis is that the SENOMIC trial protocol did not include a screening list for not included patients and therefore, the reasons for not including patients are largely unknown. The sites had a voluntary option to report reasons not to include the patient, but this was only done occasionally. Accordingly, the reasons for the different proportions of enrolled trial participants according to eligible NKBC cases are unclear. Possible explanations may be patient preference, difficulty to communicate, comorbidities not reported as well as shortage of resources and time both for informing patients and for extra follow-up, as the trial did not offer any reimbursement of expenses. Furthermore, we cannot rule out that there might have been some degree of systematic exclusion of patients in some sites.

Another limitation is that, while patient and tumor data reported in the SENOMIC trial were validated by scrutinizing every participant’s pathology reports, data from cancer registers are less rigorously managed. A validation of the NKBC in 2015, however, demonstrated an excellent data quality with a concordance between NKBC data and validation data of more than 90% [21, 24]. We therefore propose that a comparative analysis between trial data and a national register is an excellent method for external validity analyses to determine how representative patients included in a trial are for patients in the eligible background population, and how large selection effects one needs to take into account when interpreting and implementing trial results.

To our knowledge, there are no previous reports exploring the representativeness of trial data examining the oncological safety after less extensive axillary surgery. As the present comparison indicates an acceptable concordance of trial participants and NKBC population, future results from the SENOMIC trial can safely be applied to the eligible target population. However, there were small differences between the groups that one need to consider when implementing the results, not least the suggested difference in mastectomy rate. Today, the SENOMIC trial continues to include breast cancer patients with SLN micrometastases who undergo mastectomy. As the inclusion criteria have been extended to include larger tumors, the feasibility of omitting completion ALND will subsequently be clarified in further important subgroups.
